# Positive effects of prolonged caloric restriction on the population of very small embryonic-like stem cells – hematopoietic and ovarian implications

**DOI:** 10.1186/1757-2215-7-68

**Published:** 2014-06-21

**Authors:** Katarzyna Grymula, Katarzyna Piotrowska, Sylwia Słuczanowska-Głąbowska, Katarzyna Mierzejewska, Maciej Tarnowski, Marta Tkacz, Agata Poniewierska-Baran, Daniel Pędziwiatr, Ewa Suszyńska, Maria Laszczyńska, Mariusz Z Ratajczak

**Affiliations:** 1 Department of Physiology at Pomeranian, Medical University, Szczecin, Poland; 2 Department of Histology and Developmental Biology, Pomeranian Medical University, Szczecin, Poland; 3 Stem Cell Institute at the James Graham Brown Cancer Center, University of Louisville, 500 S. Floyd Street, Rm. 107, Louisville, KY 40202, USA

**Keywords:** Calorie restriction, Longevity, Bone marrow, Ovaries, Testes, VSELs

## Abstract

**Background:**

Low calorie intake, or calorie restriction (CR) without malnutrition, has been demonstrated in several animal species, including mice, to increase both median and maximum lifespan as well as delay reproductive senescence. Our previous work demonstrated a positive correlation between life span and the number of very small embryonic-like stem cells (VSELs) in long living Laron dwarf mice. These animals have very low levels of circulating insulin-like growth factor 1 (IGF-1) in peripheral blood (PB), maintain higher numbers of hematopoietic stem cells (HSPCs) in bone marrow (BM), and display prolonged fecundity compared with wild type littermates. Since CR lowers the level of IGF-1 in PB, we become interested in the effect of CR on the number of VSELs and HSPCs in BM as well as on the morphology of ovaries and testes.

**Methods:**

In our studies four-week-old female and male mice were subjected to CR by employing an alternate-day *ad libitum* feeding diet for a period of 9 months.

**Results:**

We observed that mice on CR had a higher number of BM-residing VSELs than control mice fed *ad libitum*. These changes correlated with higher numbers of HSPCs in BM, spleen, and peripheral blood (PB) as well as with an increase in the number of primordial and primary follicles in ovaries. At the same time, however, no changes were observed in the testes of mice under CR.

**Conclusion:**

We conclude that CR positively affects the pool of VSELs in adult tissues and explains the positive effect of CR on longevity.

## Introduction

One of the proposed means of increasing life span is caloric restriction (CR). In support of this notion, it has been demonstrated that CR without malnutrition is an effective means to decelerate the aging process, increase median and maximum lifespan, as well as delay reproductive senescence in a variety of species, including mice [1].

We recently reported that life span in experimental murine strains (e.g., Laron and Ames dwarf mice) correlates with the number of very small embryonic-like stem cells (VSELs) residing in adult tissues [2,3]. Specifically, long-living murine strains with low levels of insulin-like growth factor 1 (IGF-1) circulating in peripheral blood (PB) display higher numbers of VSELs in bone marrow (BM) than age-matched normal control animals [3]. The higher numbers of BM-residing VSELs in these animals also correlated with higher numbers of hematopoietic stem progenitor cells (HSPCs) in BM. This observation provides corroboration for our experimental data showing that VSELs can be specified into HSPCs and thus fulfill the criteria for long-term repopulating hematopoietic stem cells (LT-HSCs) [3].

We envision that VSELs, which express several markers of pluripotency (e.g., SSEA, Oct-4, Nanog), are a population of early-development stem cells that, due to epigenetic changes in certain paternally imprinted genes (e.g., *Igf2-H19*) involved in insulin/insulin-like growth factor signaling (IIS), are kept as a quiescent population of cells in adult tissues [2,4]. Importantly, the epigenetic mechanism that attenuates VSELs responsiveness to IIS has a positive effect on maintaining their number in adult tissues [2,4]. However, VSELS have the potential to become specified into more-differentiated tissue-committed stem cells (TCSCs) after reversing expression of imprinted genes to the somatic type [5]. We also believe that VSELs most likely overlap with other types of early-development pluri/multipotent stem cells (e.g., spore-like stem cells, multipotent adult stem cells, or multipotent adult progenitor cells) in adult tissues described by other investigators [6-8].

Interestingly, a population of small cells corresponding to BM-purified VSELs has also been described in murine ovaries and testes [9-11]. These ovary- and testis-residing VSELs have been postulated to be precursors of gametes both in mice and humans [9-11]. We observed that long-living Laron dwarf mice, which we have demonstrated to have higher numbers of VSELs in BM, have the period of active ovulogenesis prolonged to an advanced age, and Laron dwarf mice older than 2 years can become pregnant and deliver healthy offspring [12].

Based on these observations and the well-known facts that CR lowers IGF-1 levels in PB and has a beneficial effect on life span in mice, we became interested in the effect of CR on the number of murine VSELs and HSPCs as well as on the morphology of ovaries and testes. In our studies, 4-week-old female and male mice were subject to CR by permitting feeding *ad libitum* (AL) only on alternate days for a period of 9 months.

Our data indicate that mice under CR have a higher number of BM- and spleen-residing as well as PB-circulating VSELs than control mice fed AL. CR also correlated with a higher number of HSPCs in hematopoietic tissues as well as with an increase in the number of primordial and primary follicles in ovaries. At the same time, however, no significant changes were observed in the testes of mice on CR. Thus, our data explain the positive effect of CR on longevity in mice by a novel early development stem cell related mechanism.

## Material and methods

### Animals

A total of 24 four-week-old C57Bl/6 mice of both sexes were employed in our experiments. Mice were randomly divided into two groups (6 animals/group of each sex) and were housed separately in isolated cages (one mouse per cage) under controlled conditions of optimum temperature (21°C), ventilation, and light timed to follow a circadian rhythm. All animals had free access to water. One half of the mice (control) had food (Murigran, Motycz, Poland) at a recommended daily dose (7 g per mouse) *ad libitum (*AL), and the other half of the animals on CR was deprived of food and fed AL only every other day [13]. Body weight was measured once a week starting on day 0. At the end of the study (after 9 months) the mice were sacrificed. During necropsy, peripheral blood, bone marrow, spleen, testes, ovaries, heart, kidney, and brain were isolated. All animal protocols were approved by the Ethical Committee at the Pomeranian Medical University, Szczecin, Poland.

### Tissue preparation

At the end point of the experiment, bone marrow (BM), peripheral blood (PB), spleens, ovaries, testes, kidneys, hearts, and brains were isolated for histological analysis. Organs were fixed in 10%-buffered formalin for 24 hours, and after fixation, samples were dehydrated and embedded in paraffin blocks.

### Histological analysis

Deparaffinized sections of ovaries and testes (3 μm thick) and other organs were rehydrated and stained with Mayer’s hematoxylin and eosin (H&E) stain (Sigma-Aldrich, Poland) according to standard procedures. After staining, sections were dehydrated in 95% and 99.8% alcohol, cleared with xylene, mounted with Roti®-Histokitt II mounting medium, and evaluated under an Olympus IX81 inverted microscope (Olympus, Germany). Micrographs were collected with CellSens software (Olympus, Germany). The numbers of primordial, primary, pre-antral, atretic, antral and Graffian follicles in ovaries of CR mice and mice fed AL were counted by two different persons.

### Immunohistochemical (IHC) analysis of paraffin-embedded ovarian tissue

Ovarian 3-μm-thick tissue sections were deparaffinized twice in xylene (40 min and 20 min), rehydrated in alcohol (100%, 95%, 80%, and 70%), and transferred to deionized water. After antigen retrieval in a water bath containing citrate buffer pH = 6 and incubation with blocking serum, anti-growth hormone receptor (GHR) antibody was added (rabbit polyclonal anti-GHR antibody, Acris Antibodies, GmbH) at 1:250 dilution for 1 hour at room temperature. After incubation with primary antibody and washing twice in PBS, the DAB (diaminobenzidine) detection system (Dako, Denmark) was employed to visualize the immunohistochemical reaction. The sections were counterstained with Mayer’s H&E stain. Section images were acquired under an Olympus IX81 inverted microscope (Olympus, Germany), and micrographs were analyzed with CellSens software (Olympus, Germany).

### FACS purification of VSELs and HSCs from murine bone marrow and peripheral blood

The bone marrow mononuclear cells (BMMNC) necessary for FACS analysis were obtained by flushing of femurs and tibiae in a cold PBS solution containing 2% fetal bovine serum (FBS), L-glutamine, and antibiotics. After isolation, erythrocytes depleted by a 15-minute incubation in ammonium chloride-containing lysing solution (BD Pharm Lyse lysing buffer, Becton Dickinson, San Jose, CA) were passed through a 40-μm nylon mesh (Cell Strainer, BD Falcon, Becton Dickinson), centrifuged (1200 rpm at 4°C for 5 min.), and stained with antibodies as described below. Mouse peripheral blood (PB) was collected in heparinized tubes from the vena cava during sectioning. Isolation of MNCs from PB was performed in an analogous way as for bone marrow cells. VSELs and HSCs were identified in BM and PB after staining for CD45, hematopoietic lineage markers (Lin), and Sca-1 antigen expression for 30 min in medium containing 2% fetal bovine serum. The following anti-mouse antibodies (BD Pharmingen) were used for staining: rat anti-CD45 (allophycocyanin–Cy7, clone 30 F11), anti-CD45R/B220 (PE, clone RA-6B2), anti-Gr-1 (PE, clone RB6-8 C5), anti-T cell receptor-αβ(PE, clone H57-5970), anti-T cell receptor- ɤδ (PE, clone GL3), anti-CD11b (PE, clone M1/70), anti-Ter119 (PE, clone TER-119), and anti-Ly-6A/E (also known as Sca-1, biotin, clone E13-161.7, with streptovidin conjugated to PE–Cy5). Cells were then washed, resuspended in RPMI medium with 2% fetal bovine serum, and analyzed with a Navios flow cytometer (Beckman Coulter, USA). Two populations were analyzed: Lin^–^/Sca-1^+^/CD45^–^ (VSELs) and Lin^–^/Sca-1^+^/CD45^+^ (HSCs).

### Clonogenic in vitro assays

Murine HSPC’s isolated from BM, PB, and spleen were evaluated by an in vitro clonogenic assay as described previously [14]. Briefly, 2 × 10^5^ BM-derived and 4 × 10^5^ PB and spleen-derived cells were resuspended in 0.4 mL of RPMI-1640 medium and mixed with 1.8 mL of MethoCult HCC-4230 methylcellulose medium (StemCell Technologies Inc., Canada), supplemented with L-glutamine and antibiotics. Specific murine recombinant growth factors (all from R&D System, USA) were added. In the case of granulocyte-macrophage colony forming units (CFU-GM), IL-3 (20 U/mL), SCF (10 ng/mL), and GM-CSF (5 ng/mL) were used. EPO (5 U/mL) and SCF (10 ng/mL) were used for induction of erythrocyte burst-forming units (BFU-E). The colonies were counted under an inverted microscope after 7–10 days of culture. Each clonogenic test was performed in quadruplicate.

### Real-time quantitative reverse transcription PCR (RQ-PCR)

Total RNA was isolated from cells harvested from the bone marrow and peripheral blood of experimental and control mice with the RNeasy Kit (Qiagen, Valencia, CA). RNA was reverse-transcribed with MultiScribe reverse transcriptase and oligo(dT) primers (Applied Biosystems, Foster City, CA). Quantitative assessment of mRNA levels was performed by real-time reverse transcriptase polymerase chain reaction (RT-PCR) on an ABI 7500 Fast instrument using Power SyBR Green PCR Master Mix reagent. Real-time conditions were as follows: 95°C (15 sec), 40 cycles at 95°C (15 sec), and 60°C (1 min). According to melting point analysis, only one PCR product was amplified under these conditions. The relative quantization value of target (the fold change) normalized to the endogenous control β-2 microglobulin gene and relative to a calibrator is expressed as 2^∆∆Ct^, where ∆Ct = (Ct of target genes) – (Ct of endogenous control gene, β-2 microglobulin), and ∆∆Ct = (∆Ct of sample for target gene) – (∆Ct of calibrator for the target gene). The following primer pairs were used: β-2 microglobulin (forward): 5′-CAT ACG CCT GCA GAG TTA AGC A-3′; β-2 microglobulin (reverse): 5′-GAT CAC ATG TCT CGA TCC CAG TAG-3′; Oct-4 (forward): 5′- ACA TCG CCA ATC AGC TTGG -3′; Oct-4 (reverse): 5′-AGA ACC ATA CTC GAA CCA CAT CC -3′; Nanog (forward): 5′-TTT TCA GAA ATC CCT TCC CTC G -3′; Nanog (reverse): 5′-CGT TCC CAG AAT TCG ATG CTT -3′.

### Statistical analysis

All results are presented as mean ± SD. Statistical analysis of the data was done using Student's *t*-test for unpaired samples, with p < 0.05 considered significant.

## Results and discussion

Calorie restriction (CR) without malnutrition significantly increases life span in various species, ranging from yeast to primates, including laboratory rodents [15]. It has also been demonstrated that CR maintains tissues in a youthful state, and in many species studied so far, delays reproductive senescence [1]. Several experimental protocols have been proposed for maintaining mice on CR for different periods of time at different ages [16-24].

In our work, to study the effect of CR on the populations of VSELs and HSPCs and the morphology of major organs, including gonads, 4-week-old female and male C57Bl6 mice (6 animals/sex/group) were subjected to CR by employing an *ad libitum* (AL) feeding diet every other day for a period of 9 months [13]. In parallel, control female and male mice (6 animals/sex/group) received food AL every day. At the end of the experiment, animals were sacrificed and i) peripheral blood (PB) was drawn for blood count analysis, ii) bone marrow (BM) was isolated from long bones to enumerate the number of Sca-1^+^lin^–^CD45^–^ (VSELs) and Sca-1^+^Lin^–^CD45^+^ (HSPCs) by FACS, iii) the clonogenic potential of HSPCs in vitro was evaluated by colony-forming assays, and iv) the mRNA levels for selected pluripotency markers (Oct-4 and Nanog) were determined. We also harvested brain, heart, and kidneys from all animals as well as ovaries and testes for histological analysis.

We observed that the CR feeding protocol had a different impact on average body weight between male and female mice (Additional file 1: Figure S1). While male mice on CR were leaner and had an ~25% reduction of body weight compared with normal littermates fed AL, female animals maintained a similar body weight in both the CR and AL groups. This may be explained by the possibility that in female mice, the lack of the food intake was compensated every second day by increased food intake when it was available AL. This “compensatory” feeding behavior of female mice compared with males is intriguing and requires further study. We can not also exclude an alternative explanation that female mice exert-hormone dependent slower metabolism rate and/or activity pattern as compared to male mice.

Our analysis of CR- and AL-fed control mice revealed that the peripheral blood red blood cell and leukocyte counts as well as hematocrit, mean corpuscular volume (MCV), mean corpuscular hemoglobin (MCH), and mean corpuscular hemoglobin concentration (MCHC) values were not statistically different between groups of mice, regardless of their sex (Additional file 2: Figure S2). We also did not detect any differences in the morphology of brain, heart, and kidneys between experimental and control mice (data not shown). Nevertheless, it is well known that CR has a beneficial effect on age-related cardiovascular and neural complications and also has an anti-cancer effect. Since we terminated our experiments when animals were ~1 year of age (10 months), we assume that more sensitive assays could be employed to detect potential morphological changes, and, most likely, such changes would have occurred if we had kept our animals for a longer period of time before analysis.

**Figure 1:**
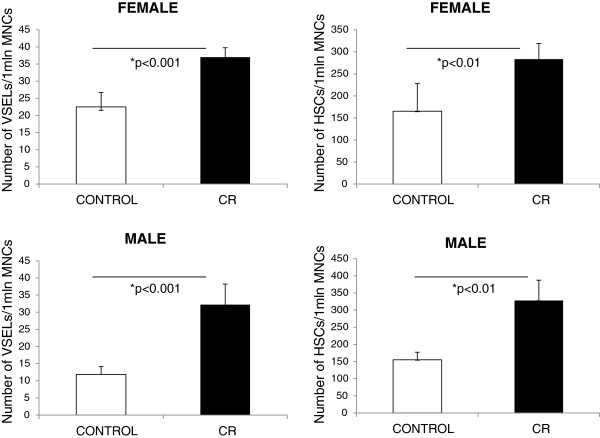
**Increases in the number of stem cells in the BM of 10-month-old female and male C57B1/6 mice on CR compared with controls fed AL.** The number of Sca-1^+^Lin^–^CD45^–^ VSELs (**left panels**) and Sca-1^+^Lin^–^CD45^+^ HSPCs (**right panels**) in the BM of mice on CR or of control mice fed AL (6 mice/group).

However, as shown in Figure 1, our FACS analysis of the number of VSELs and HSPCs in the BM of female (upper panel) and male (lower panel) mice after 9 months on CR revealed a significant increase in the numbers of both types of stem cells in both sexes compared with animals on an AL feeding regimen. These observations demonstrate for the first time the beneficial effect of CR on the number of BM VSELs. It also supports observations from other teams that CR has a beneficial effect on tissue-committed stem cells, including HSPCs in BM [25], as well as skeletal muscle [26] and neural [27] stem cells. However, we must point out that in these cited studies different strains of mice were employed, different CR protocols were used, and the animals were analyzed at different ages [25-27]. Nevertheless, our study is in agreement with the conclusion of the abovementioned studies [25-27] that CR has a protective effect on the stem cell compartment.

We have proposed that VSELs in adult tissues are somewhat protected by maternal imprinting at developmentally/metabolically important loci that attenuates excessive insulin/insulin-like growth factor signaling (IIS). Since CR lowers the levels of insulin, growth hormone, and IGF-1 in peripheral blood, this effect may somewhat protect VSELs from premature depletion in adult tissues. In support of this possibility, we have demonstrated that long-living Laron and Ames dwarf mice, with very low levels of circulating IGF-1, have higher numbers of VSELs in BM at advanced ages than wild type littermates [3]; moreover, female Laron dwarf mice >2 years of age still exhibit active ovulogenesis and can deliver viable offspring [12].

As has been reported, VSELs are precursors of long-term repopulating hematopoietic stem cells (LT-HSCs) in BM [14,28]. In agreement with this observation, long-living Laron and Ames dwarf mice, which have higher numbers of VSELs in BM, also have higher numbers of HSPCs at advanced ages than their wild type littermates [3]. The conclusion of these studies was that VSELs are a quiescent population of cells in adult BM, and their proliferation state is regulated by maternal imprinting at genes regulating IIS signaling [4,14]. This has been recently confirmed by another team showing that murine LT-HSCs are maintained in a quiescent state by maternal imprinting at the IGF2-H19 locus [29].

**Figure 2:**
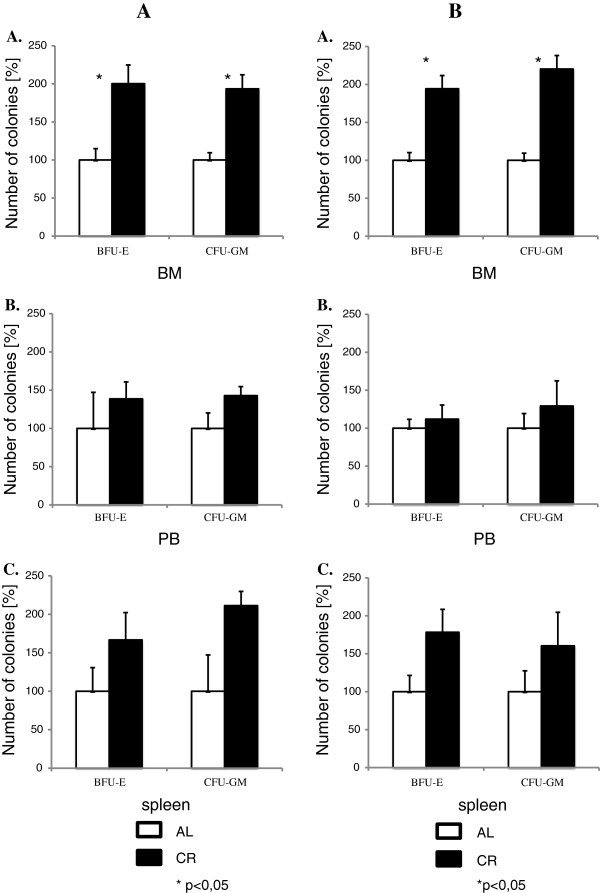
**The number of clonogenic BFU-E and CFU-GM in hematopoietic tissues of 10-month-old female (panel A) and male (panel B) C57B1/6 mice on CR or fed AL.** Mononuclear cells from bone marrow **(A)**, peripheral blood **(B)**, or spleen **(C)** from mice (6 animals/group) were plated in vitro in methylcellulose cultures to evaluate the number of clonogenicerythroid BFU-E and myeloid CFU-GM progenitors. The number of colonies grown from mice fed AL is shown as 100%.

Changes in the number of VSELs and HSPCs in BM reported herein were correlated with an increase in the number of clonogenic progenitors for both myeloid (CFU-GM) and erythroid (BFU-E) lineages in female (Figure 2A) and male (Figure 2B) mice in BM (panels A), PB (panels B), and spleen (panels C). These observations further support a link between VSELs and LT-HSCs [28]. Similarly, a beneficial effect on the number of clonogenic HSPCs circulating in PB has been reported recently in patients [30]. Moreover, an increase in the number of VSELs in BM observed by FACS corresponded with an increase in the expression level of mRNA for Oct-4 and Nanog, which are known transcription factors involved in the development of pluripotent stem cells and are expressed in VSELs (Additional file 3: Figure S3).

**Figure 3:**
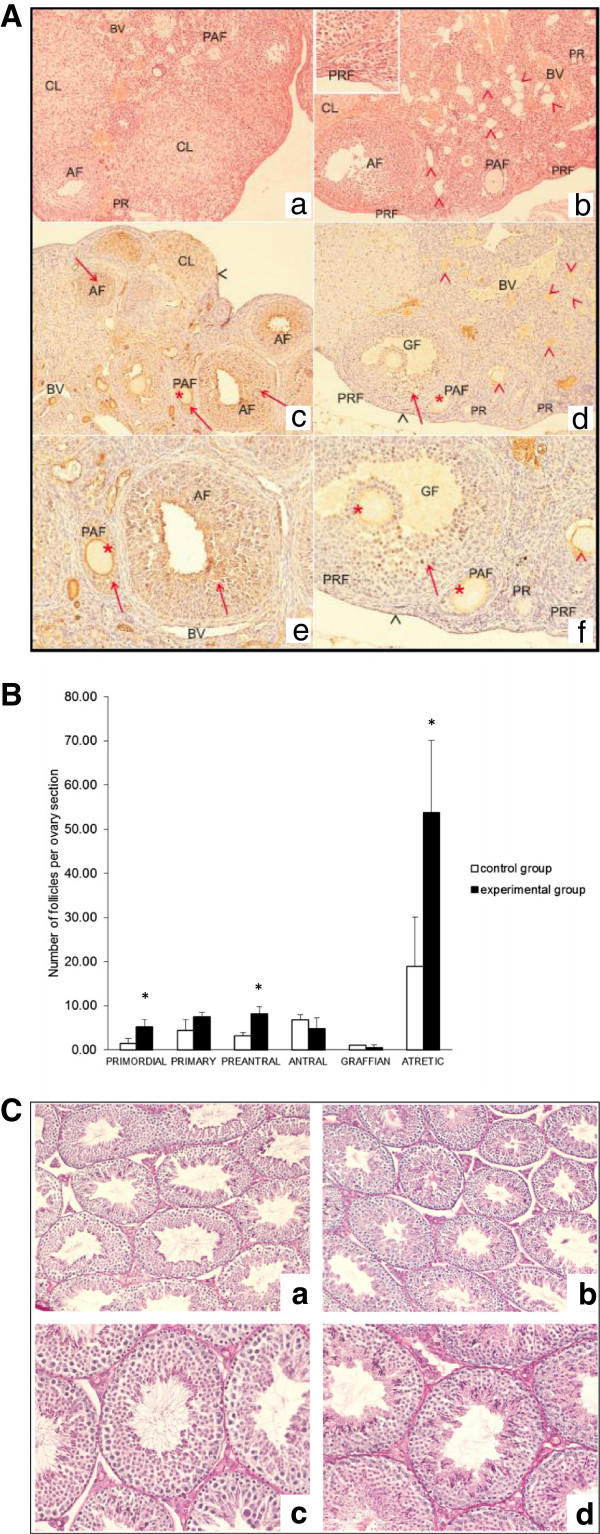
**Effect of CR on morphology of ovaries and testes.** Panel **A**. Ovaries of 10-month-old C57B1/6 mice fed AL (a, c, and e) or on CR (b, d, and f). a and b. In both groups, primary (PF), preantral (PAF), and antral (AF) follicles and corpus luteum (CL) are visible. We also observed primordial follicles and a greater number of atretic follicles (red arrowheads) in the CR group (b) than in the AL-fed group (a). Hematoxylin + eosin staining. Magnification: x100. Inset in panel b represents primordial follicles in an ovary from a mouse on CR. Magnification, x400. c-f. Immunohistochemical reaction for growth hormone receptor (GHR) expression in ovaries of mice fed AL (c and e) or on CR (d and f). High expression of GHR in *ad libidum*(AL)-fed mice was noted in granulosa cells (red arrow), preantral and antral follicles and zona pellucida (asterisk), ovarian surface epithelium (black arrowhead), and blood vessel endothelium (BV, c and e). By contrast, expression of GHR in CR mice was found only in a few granulosa cells (red arrow) of Graffian follicles (GF), and no expression of GHR was observed in granulosa cells of preantral (PAF), primary (PF), and primordial (PRF) follicles (d and f). Magnification: panels c and d, x100; panels e and f, x200. Representative images are shown. Panel **B**. The number of follicles at different stages of development in ovaries of 10 month-old female C57B/6 mice on CR compared with controls fed AL. The higher number of primordial, primary, preantral and atretic follicles in ovaries of mice on CR than in ovaries of mice fed ad libidum. P < 0.01. Panel **C**. Testes of 10-month-old C57B1/6 mice fed AL (panels a and c) or on CR (panels b and d). Magnification: panels a and b, x100; panels c and d, x200. PAS staining. Representative images are shown.

Since positive effects of CR on fertility and the germ line compartment in gonads in different species have been reported [1], we focused on the morphology of ovaries in our CR- and AL-fed mice (Figure 3). Careful histological analysis revealed that ovaries from CR female mice (Figure 3A) possess an advantage in reproductive potential compared with control animal ovaries. Specifically, in the ovaries of mice fed AL, we noted smaller numbers of primordial, primary, and, preantral follicles than in the ovaries of mice on CR (Figure 3A and B). Histochemical reaction for growth hormone receptor (GHR) was found in ovaries of mice fed AL in granulosa cells, zona pellucida, ovarian surface epithelium, and in blood vessel epithelium (Figure 3A). Moreover, these mice had a positive reaction for GHR in granulosa cells in all stages of follicular development (primary, preantral, antral, and Graffian follicles), except in granulosa cells of primordial follicles. By contrast, mice on CR had a positive reaction for GHR only in a few granulosa cells in Graffian and antral follicles and lacked expression of GHR in granulosa cells in primordial, primary, and preantral follicles.

Growth hormone (GH) is a potent activator of growth and development of ovarian follicles and also suppresses apoptosis and atresia in rodent ovaries [31]. Our results show that CR leads to a decrease in expression of GHR in granulosa cells, ovarian surface epithelium, and in zona pellucida, particularly at younger stages of follicular development. This decrease in the expression of its receptor leads to a decrease in the effect of GH at the early stages of follicle development, which results in a greater number of primordial and primary follicles in the ovaries of mice on CR. Based on these changes, mice on CR possess an advantage in reproductive potential compared with control animals. Thus, our data obtained in mice fed every other day (even if they maintained a similar weight as control AL animals) are in agreement with an earlier study where female animals on continuous CR received fewer calories every day [1]. These observations have important clinical implications, and it is well known that one of the strategies for increasing female fertility in humans is CR [32].

These data are also important from a different perspective. It has been reported that adult ovaries and testes contain a population of VSELs that could be precursors of gametes [9-11], and 2- year-old Laron dwarf mice, which maintain a high number of VSELs in tissues, have prolonged fertility, as mentioned above [12]. Therefore, further studies are needed to evaluate whether CR directly affects, in a positive way, the pool of VSELs in murine ovaries [9-11]. Interestingly, at the same time we did not observe significant changes in the testes of animals on CR. By way of explanation, it is known that senescence in the male reproductive system is less dramatic over time than that of females. Thus, more sensitive detection methods and more work on mice on CR for longer periods would probably be needed to detect differences between CR and control male mice.On the other hand, the reproductive success of female mice depends on long periods of pregnancy and feeding of offspring, and CR might directly impair these aspects of reproduction. However, under CR the ovaries could maintain the pool of small undeveloped (primordial) follicles, waiting for improvement in environmental conditions that will better guarantee reproductive success. By contrast, males produce more sperm cells than needed for reproduction, and thus even a significant decrease in sperm count will not impair reproductive potential in a dramatic way. In addition, male involvement in reproduction is less time- and energy-consuming than for females, and CR should not affect testes as much as ovaries. This may explain at least partly why we did not noticed significant morphological changes in testes of males on CR as compared to mice fed AL (Figure 3C).

In conclusion, our data support a potential role for CR in maintaining the pool of VSELs in regulating life span in normal mice and indicate that pharmacological strategies aimed at maintaining higher numbers of these cells in adult tissues, as manipulated here by CR, may have beneficial effects on the extension of life span. We envision that CR may facilitate a potential role these cells are playing in tissue organ rejuvenation as a source of tissue specific stem cells (e.g., neural stem cells, skeletal muscle stem cells). VSELs have been successfully isolated in several laboratories and have been demonstrated to differentiate into cells from all three germ layers [33-36]. Further studies are needed to shed more light on CR and their role in longevity in humans.

## Competing interests

The authors declare that they have no competing interests.

## Authors’ contributions

KG carried out the study, collected material, performed the clonogenic assays, KP participated in study, collection of the material, prepared histological samples, analyzed histological and IHC data, participated in preparation of manuscript, SS-G prepared histological samples analyzed histological and IHC data, KM performed cytometric analysis of cells, participated in collection of the material, MT performed genetic analysis, MT participated in material collection and in clonogenic assays, AP-B participated in material collection and genetic analysis, DP participated in cytometric analysis, ES participated in cytometric analysis, ML analyzed histological and IHC data, participated in preparation of manuscript, MZR designed and coordinated the study, wrote the manuscript and provided funding. All authors read and approved the final manuscript.

## Supplementary Material

Additional file 1: Figure S1 Average weight of male (panel A) and female (panel B) mice on CR or fed AL. Click here for file

Additional file 2: Figure S2 Peripheral blood counts of mice at CR and fed AL. Click here for file

Additional file 3: Figure S3 Real time expression analysis of Oct-4 and Nanog expression in bone marrow mononuclear cells (BMMNC). BMMNC were isolated from 10 month old C57B1/6 mice fed ALand mice on CR. Expression of Oct-4 and Nanog in mice fed AL was assumed to be 1.0. There were analyzed 6 mice/group. Combined data are presented Panel A: Females; Panel B: Males. *p < 0.05. Click here for file
