# Supplementary material for: Positive effects of prolonged caloric restriction on the population of very small embryonic-like stem cells – hematopoietic and ovarian implications
Source: J Ovarian Res. 2014 Jun 21;7:68. doi: 10.1186/1757-2215-7-68 (PMC4076763; doi:10.1186/1757-2215-7-68)
Supplement: Additional file 1: Figure S1 — Average weight of male (panel A) and female (panel B) mice on CR or fed AL. [file 1757-2215-7-68-S1.ppt]

## Slide 1
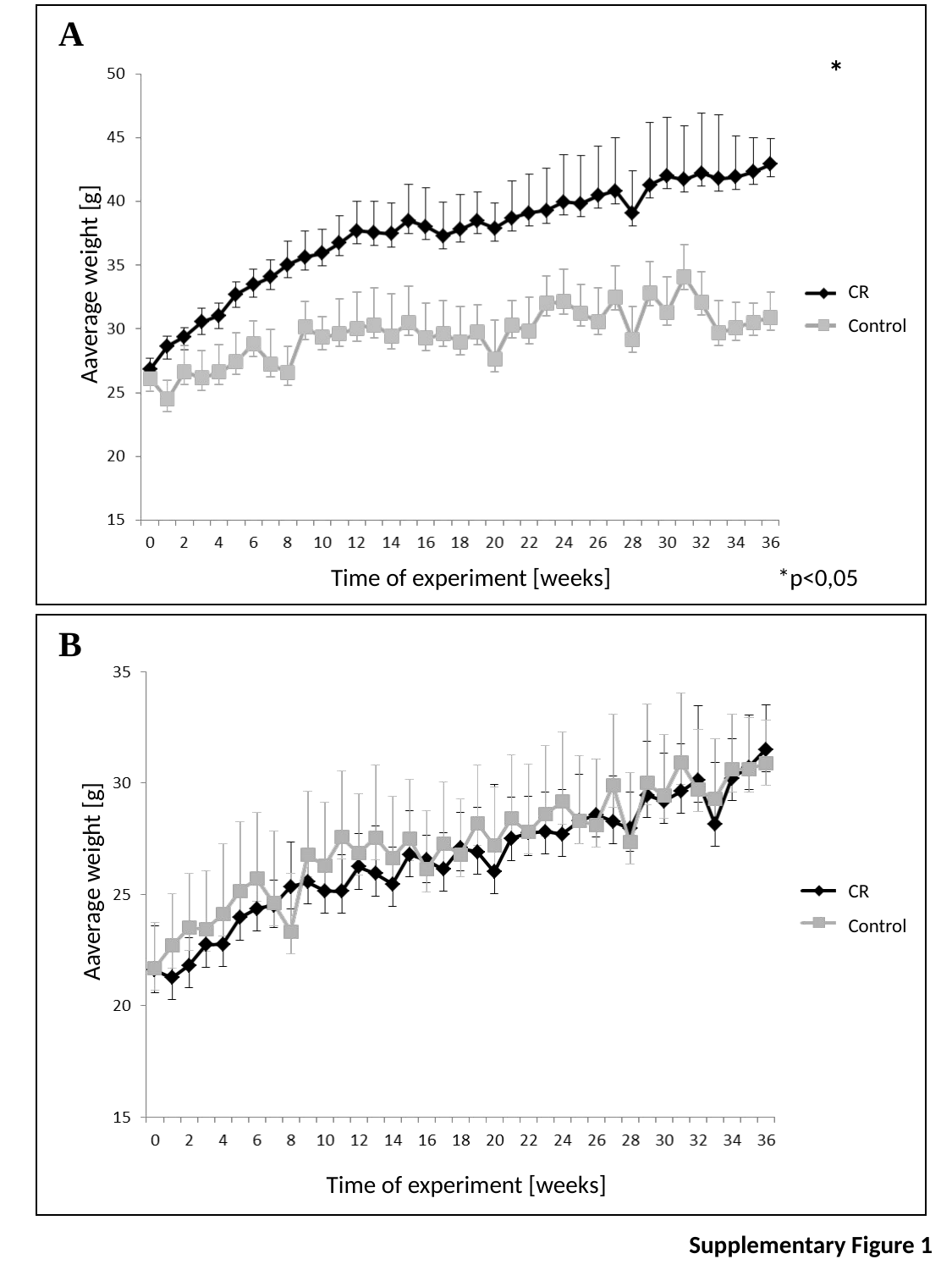

A
*
Aaverage weight [g]
CR
Control
Time of experiment [weeks]
*p<0,05
B
Aaverage weight [g]
Time of experiment [weeks]
CR
Control
 Supplementary Figure 1
