# Supplementary material for: Positive effects of prolonged caloric restriction on the population of very small embryonic-like stem cells – hematopoietic and ovarian implications
Source: J Ovarian Res. 2014 Jun 21;7:68. doi: 10.1186/1757-2215-7-68 (PMC4076763; doi:10.1186/1757-2215-7-68)
Supplement: Additional file 2: Figure S2 — Peripheral blood counts of mice at CR and fed AL. [file 1757-2215-7-68-S2.ppt]

## Slide 1
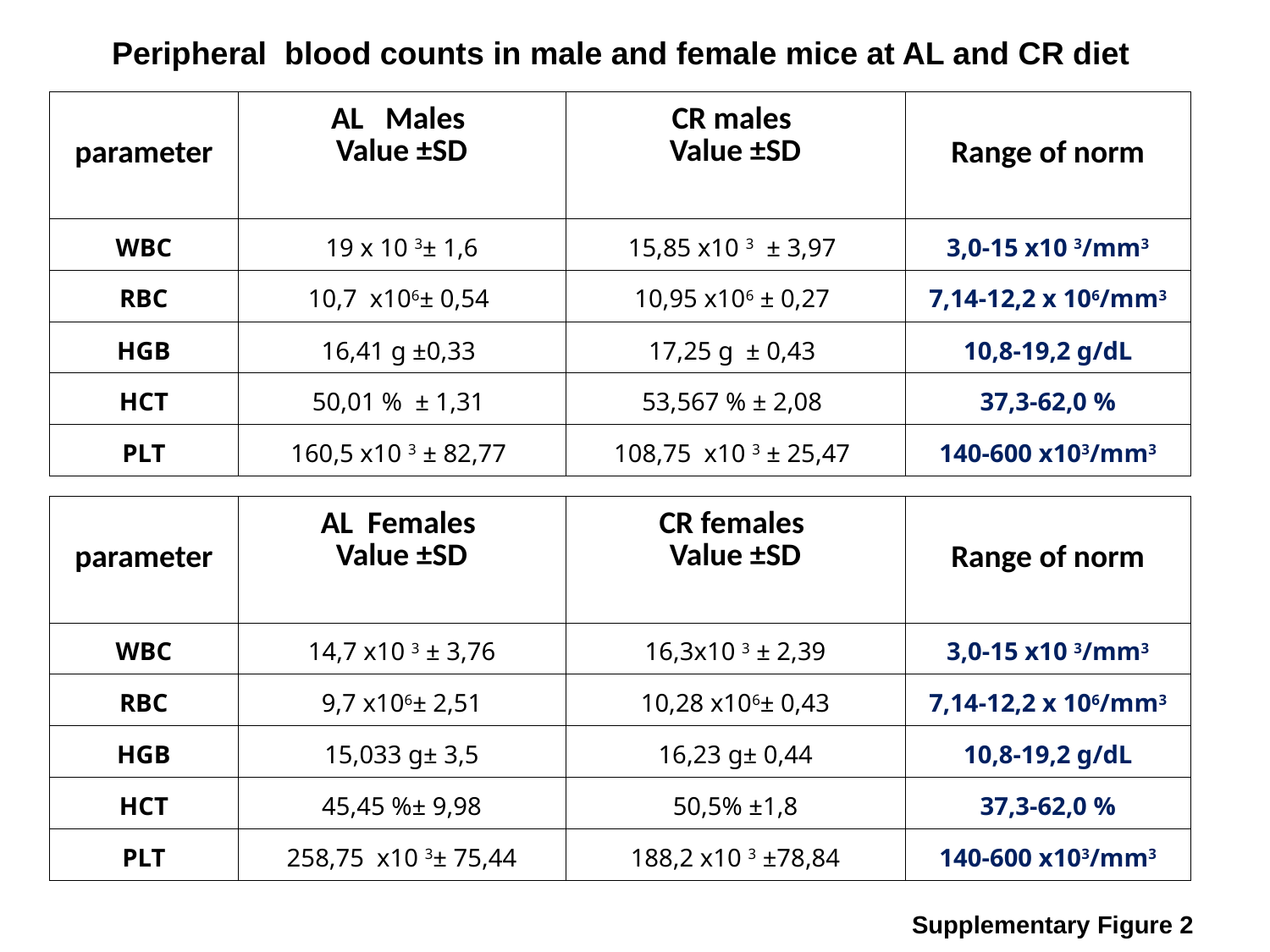

Peripheral blood counts in male and female mice at AL and CR diet
| parameter | AL Males Value ±SD | CR males Value ±SD | Range of norm |
| --- | --- | --- | --- |
| WBC | 19 x 10 3± 1,6 | 15,85 x10 3 ± 3,97 | 3,0-15 x10 3/mm3 |
| RBC | 10,7 x106± 0,54 | 10,95 x106 ± 0,27 | 7,14-12,2 x 106/mm3 |
| HGB | 16,41 g ±0,33 | 17,25 g ± 0,43 | 10,8-19,2 g/dL |
| HCT | 50,01 % ± 1,31 | 53,567 % ± 2,08 | 37,3-62,0 % |
| PLT | 160,5 x10 3 ± 82,77 | 108,75 x10 3 ± 25,47 | 140-600 x103/mm3 |
| parameter | AL Females Value ±SD | CR females Value ±SD | Range of norm |
| --- | --- | --- | --- |
| WBC | 14,7 x10 3 ± 3,76 | 16,3x10 3 ± 2,39 | 3,0-15 x10 3/mm3 |
| RBC | 9,7 x10^6^± 2,51 | 10,28 x106± 0,43 | 7,14-12,2 x 106/mm3 |
| HGB | 15,033 g± 3,5 | 16,23 g± 0,44 | 10,8-19,2 g/dL |
| HCT | 45,45 %± 9,98 | 50,5% ±1,8 | 37,3-62,0 % |
| PLT | 258,75 x10 3± 75,44 | 188,2 x10 3 ±78,84 | 140-600 x103/mm3 |
 Supplementary Figure 2
