# Supplementary figures and images for: Positive effects of prolonged caloric restriction on the population of very small embryonic-like stem cells – hematopoietic and ovarian implications
Source: J Ovarian Res. 2014 Jun 21;7:68. doi: 10.1186/1757-2215-7-68 (PMC4076763; doi:10.1186/1757-2215-7-68)

## Slide 1
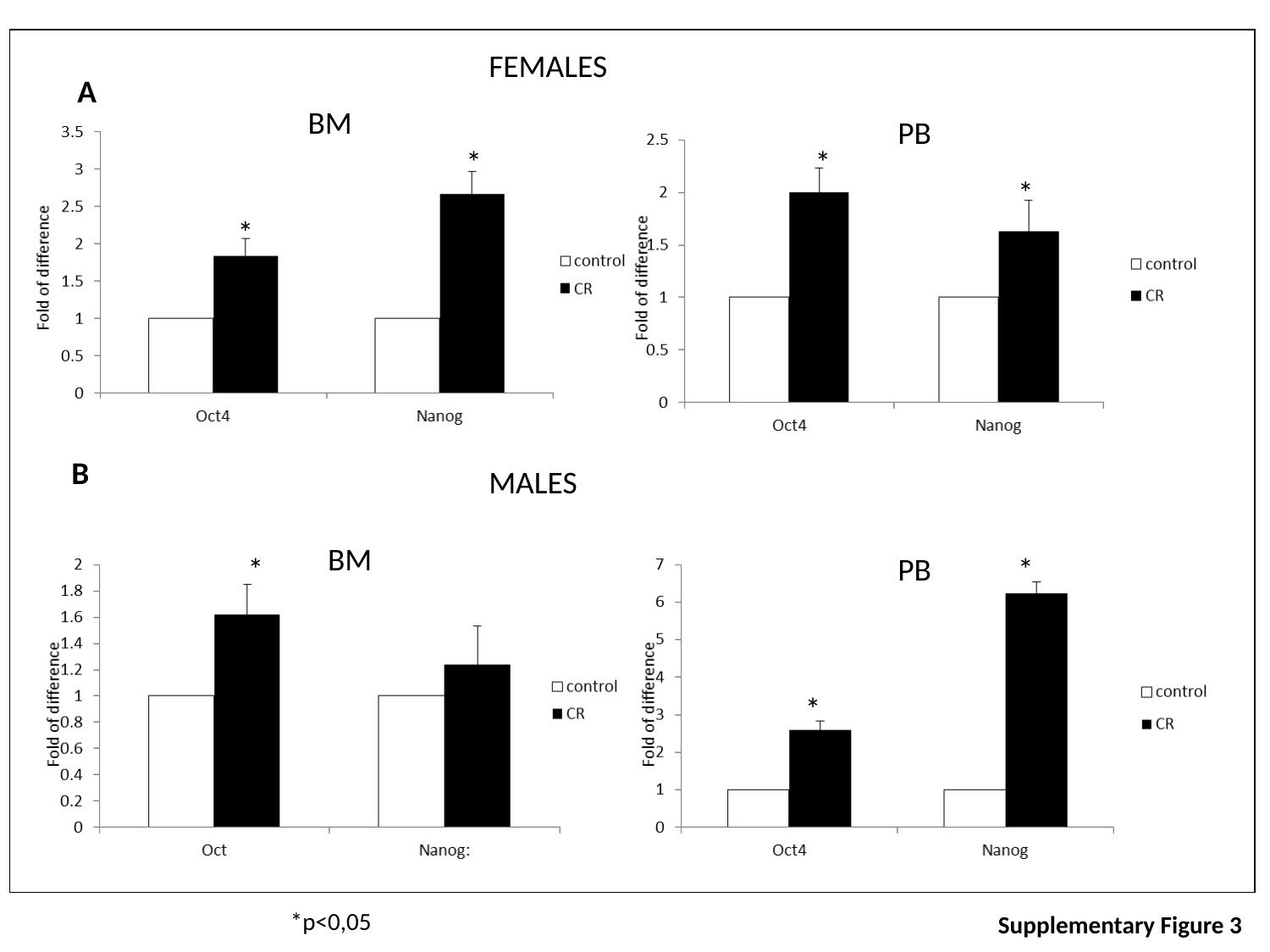

FEMALES
A
BM
PB
*
 *
*
*
B
MALES
BM
*
PB
*
 *
 Supplementary Figure 3
*p<0,05

Supplement: Additional file 3: Figure S3 — Real time expression analysis of Oct-4 and Nanog expression in bone marrow mononuclear cells (BMMNC). BMMNC were isolated from 10 month old C57B1/6 mice fed ALand mice on CR. Expression of Oct-4 and Nanog in mice fed AL was assumed to be 1.0. There were analyzed 6 mice/group. Combined data are presented Panel A: Females; Panel B: Males. *p < 0.05. [file 1757-2215-7-68-S3.ppt]
